# THE LOOMING POST-ACUTE REORGANIZATION: IMPLICATIONS FOR OLDER ADULTS TRANSITIONING FROM THE HOSPITAL

**DOI:** 10.1093/geroni/igac059.725

**Published:** 2022-12-20

**Authors:** Taylor Bucy, John McHugh, Dori Cross

**Affiliations:** University of Minnesota School of Public Health, Minneapolis, Minnesota, United States; Columbia University Mailman School of Public Health, New York, New York, United States; University of Minnesota School of Public Health, Minneapolis, Minnesota, United States

## Abstract

Post-acute volume plays an important role in the care of millions of older adults transitioning from a hospital stay. To help reduce the total cost of care, many hospitals have been shifting more volume from institutional skilled nursing care to less expensive home health care. In fact, in 2017, home health volume eclipsed skilled nursing facility volume as the preferred post-acute destination for the first time. The COVID-19 pandemic further exacerbated this trend with many older adults preferring to avoid institutional care. Using 2016-2019 MedPAR data, we explored changes over time in hospital discharges to skilled nursing facilities versus to home health. We regress the ratio of home health to skilled nursing facility volume on a month-year variable. We find that the ratio of discharges to home health versus to skilled nursing facility increases by .07 percentage points per month (coefficient = .00073, 95% CI [.00063 to .00084]). This translates to a nearly one percentage point change in the ratio of home health to skilled nursing volume per year. These trends vary by patient characteristics (e.g. hospital diagnosis), organizational characteristics (e.g. hospital market relationships), and indicators of market capacity of post-acute services. This growing trend has implications on vulnerable older adults; health system leaders need policy guidance and incentives to invest in value-oriented care practices across this changing post-acute landscape.

